# Intrinsically disordered regions are not sufficient to direct the compartmental localization of nucleolar proteins in the nucleus

**DOI:** 10.1371/journal.pbio.3002378

**Published:** 2023-11-09

**Authors:** Emily D. Lavering, Maunika Gandhamaneni, Daniel L. Weeks

**Affiliations:** 1 Biochemistry and Molecular Biology Department, Carver College of Medicine, University of Iowa, Iowa City, United States of America; 2 Chemical Engineering, University of Iowa, Iowa City, United States of America; National Cancer Institute, UNITED STATES

## Abstract

The nucleolus is a non-membrane bound organelle central to ribosome biogenesis. The nucleolus contains a mix of proteins and RNA and has 3 known nucleolar compartments: the fibrillar center (FC), the dense fibrillar component (DFC), and the granular component (GC). The spatial organization of the nucleolus is influenced by the phase separation properties of nucleolar proteins, the presence of RNA, protein modification, and cellular activity. Many nucleolar proteins appear to concentrate within the borders of the compartments. We investigated whether the intrinsically disordered regions from several proteins provided the information needed to establish specific compartment localization using *Xenopus laevis* oocytes. For the proteins we tested, the disordered regions were not sufficient to direct specific domain localization and appear dispensable with respect to compartmentalization. Among the proteins that colocalize to the DFC are the quartet that comprise the box H/ACA pseudouridylation complex. In contrast to the insufficiency of IDRs to direct compartment localization, we found that the DFC accumulation of 2 box H/ACA proteins, Gar1 and Nhp2, was disrupted by mutations that were previously shown to reduce their ability to join the box H/ACA complex. Using a nanobody to introduce novel binding to a different DFC localized protein, we restored the localization of the mutated forms of Gar1 and Nhp2.

## Abstract 2

How do proteins sort into non-membrane bound compartments like the nucleolus? This study shows that while for some proteins it starts with having an intrinsically disordered domain, enhanced localization may require being part of larger complex.

## Introduction

**Fig 1:**
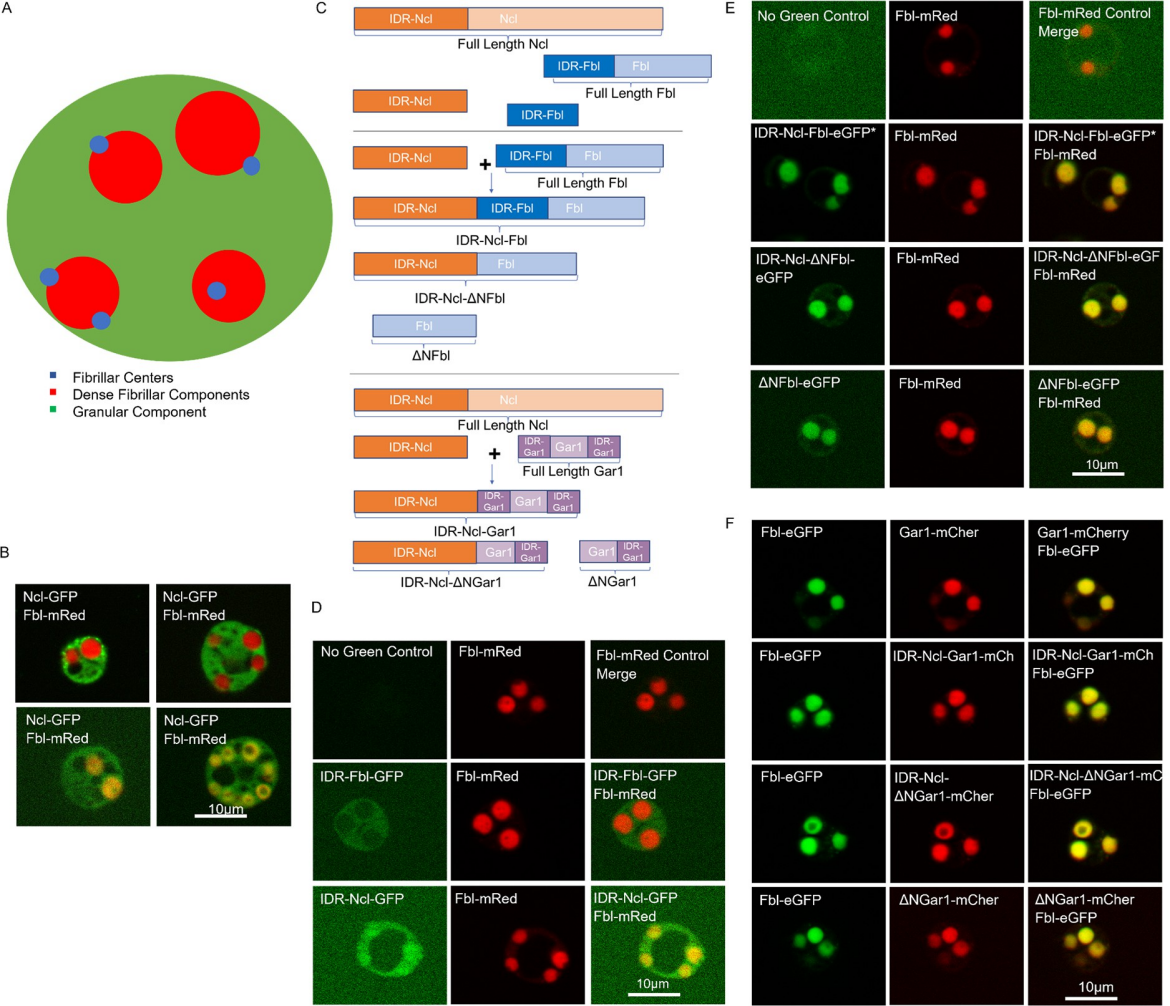
IDRs in nucleolar localization. **(A)** Model of the 3 nucleolar subdomains or compartments. **(B)** Biological variation of wild-type Ncl-GFP co-expressed with Fbl-mRed, shown as expressed in four different frogs, showing that Ncl is always in the GC and sometimes spreads into the DFC. **(C)** Schematic of the protein fusions shown in D-F. Each of these fusions also contains a green or red fluorescent protein as indicated in B that allowed detection. The IDR-Ncl has a net negative charge of −40, the IDR-Fbl has a net positive charge of +17, and the N terminal IDR-Gar1 has a net positive charge of +7. **(D–F)** Representative images of nucleoli with IDR chimera proteins fused with a fluorescent protein co-expressed with a DFC nucleolar domain marker (Fbl). **(D)** Representative images showing the intrinsically disordered regions of Fbl (IDR-Fbl-GFP) and Ncl (IDR-Ncl-GFP) co-expressed with Fbl-mRed. **(E)** Representative images showing the fusions of IDR-Ncl and Fbl, showing IDR-Ncl-Fbl-eGFP, IDR-Ncl-ΔNFbl-eGFP, and ΔNFbl-eGFP all co-expressed with Fbl-mRed. *IDR-Ncl-Fbl-eGFP images were taken at a lower exposure in the green channel due to it being far brighter than the other constructs in this set. **(F)** Representative images showing the fusions of IDR-Ncl and Gar1, showing the localization of Gar1-mCherry (WT that localizes to the DFC), IDR-Ncl-Gar1-mCherry, IDR-Ncl-ΔNGar1-mCherry, and ΔNGar1-mCherry all co-expressed with Fbl-eGFP. Scale 10 μm. Statistical analysis is shown in S1 Fig and S2 Data. DFC, dense fibrillar component; IDR, intrinsically disordered region.

The nucleolus is a prominent non-membrane-bound organelle within the nucleus that contains multiple phase-separated compartments. *Xenopus laevis* oocyte nucleoli have 3 identifiable nucleolar domains: fibrillar centers (FCs), dense fibrillar components (DFCs), and a granular component (GC) as is modeled in Fig 1A [1,2]. These 3 compartments have been described numerous times in human cells, though more recently, the proteome for a fourth compartment, the periphery of the dense fibrillar component (PDDF) was proposed as an additional distinct regions between the DFC and the GC in human cells [3]. In eukaryotes, the nucleolus serves as the center of ribosome biogenesis. The compartments of the nucleolus mediate sequential roles in ribosome biogenesis, starting with rDNA transcription, which occurs at the border between the FC and DFC, and progressing as pre-rRNA is modified in the DFC, followed by ribosomal assembly and processing in the GC [1,4,5]. Fibrillarin (Fbl) and Nucleophosmin (Npm1) are canonical domain markers for the DFC and the GC, respectively; both proteins have been shown to phase-separate in vitro and in vivo [6,7]. Fbl and Npm1 play successive roles in ribosome biogenesis, Fbl as part of a pre-rRNA methylation complex and Npm1 in ribosomal protein assembly and transport [8,9].

While the phase separation between Fbl and Npm1 has previously been characterized [6], they are only 2 of the hundreds of proteins that can be found in the nucleolus. Other potential condensate-forming nucleolar proteins were identified in *Xenopus* nuclei in previous studies by Hayes and colleagues, who generated a roster of proteins that were diffusion limited when soluble proteins were depleted from nuclei in *Xenopus laevis* oocytes [7]. Many proteins from this roster overlap with other protein condensate studies done both in vitro and in vivo [6,10,11]. Although other non-membrane bound condensates may have multiple, separate domains, the compartments in the nucleolus are especially distinct. *Xenopus laevis* oocytes are a particularly good system in which to study the nucleolus due to their large size (the diameter of a *Xenopus* oocyte nucleolus is 5–10 μm, for comparison the diameter of an entire HeLa cell is 10–40 μm), which allows for high-resolution visualization of nucleolar domains in unfixed samples using confocal fluorescent microscopy. Additionally, to account for the increased number of ribosomes needed as the oocyte prepares for embryogenesis, *Xenopus laevis* oocytes contain hundreds of nucleoli formed around extra-chromosomal rDNA.

The formation of protein condensates through phase separation is affected by many factors, including protein concentration, post-translational modifications, the presence of chaperones and/or small molecules such as ATP, and the availability of multivalent interactions [7,12,13]. Here, we investigate several features of nucleolar proteins that may contribute to specific nucleolar compartment localization. We examine if the prevalence of positive or negative charge in the intrinsically disordered regions of a protein, or an ability to bind to other proteins in a mixed protein complex is sufficient to direct compartment localization. We find that for the proteins tested, intrinsically disordered regions are not sufficient to direct compartmentalization within the nucleolus.

## Results

### Intrinsically disordered regions and nucleolar domain localization

One of the roles played by intrinsically disordered regions (IDRs) present in proteins is to drive condensate and/or aggregate formation [6,14,15]. These regions often have extreme amino acid bias and are also referred to as low complexity domains. Removal or interruption of these IDRs in some phase-separating proteins prevents condensation [6,10,16–18]. For example, the removal of IDRs from Npm1 and Fbl prevents their phase separation in vitro [6]. Additionally, some IDRs, when separated from the rest of the full-length protein, are themselves able to phase separate [6,19]. Among the proteins identified by Hayes and colleagues as forming condensates or being condensate associated in *Xenopus* oocyte nuclei, were more than 20 proteins involved in ribosome biogenesis [11]. We verified the DFC localization of 6 different nucleolar proteins and the GC localization of 8 proteins using fluorescent protein fusions [5]. We noted that some proteins appear to have sharp compartmental borders while others concentrated in one compartment but were detected throughout the nucleolus. The proteins with verified compartmentalization were examined using the program Pondr-Fit, a meta-predictor of intrinsically disordered amino acids, to predict their IDRs (S1 Table) [5,20]. These regions were also predicted using AlphaFold2 by visualizing their structures [21]. Four proteins that form parts of 2 different rRNA modification complexes, Fbl, Nop56 (box C/D modification complex), and Gar1 and Dkc1(box H/ACA complex) have IDRs with positive charges, while 3 other DFC localizing proteins that are part of the same complexes are nearly neutral. Three predominately GC localizing proteins, Npm1, Bop1, and Npm3 have IDRs with a negative charge of −19 or greater while 2 others, Pes1 and Rpl12 had more modest negative charges. Two GC localizing proteins reported to be involved in large ribosomal subunit assembly, Gtpbp4 and Pak1ip, have IDRs with positive charges. Ncl provides an interesting case, having an N terminal IDR that is very negative and C terminal IDR that is positive. Ncl can be found in both the GC and the DFC, with a slight preference for the GC. The emerging trend is that GC-localizing proteins involved in shuttling and pre-RNA processing had IDRs with significant negative charges, while the DFC proteins (all involved in rRNA modification) tended to have IDRs with positive charges.

### IDRs from Ncl, Fbl, and Gar1 are not sufficient to direct domain specificity

To determine if the presence of charged IDRs is sufficient to drive localization to a subdomain of the nucleolus, we examined the highly negative IDR from Ncl and the positively charged IDRs from Fbl and Gar1. Fbl is part of the box C/D methylation complex and Gar1 is part of the box H/ACA pseudouridylation complex. Both Fbl and Gar1 localize to the DFC (Fig 1). The experimental design used throughout this manuscript observed fluorescently labeled proteins of interest (with specified alterations), generated through mRNA injection, which join their endogenous counterparts in pre-established nucleoli. In our studies, we note some frog-to-frog variation in the precise pattern of Ncl localization, but Ncl is generally more prevalent in the GC, though not excluded from the DFC (Figs 1B and S1A). Ncl has roles in ribosome assembly and pre-rRNA transcription consistent with a broader distribution in the nucleolus [22,23].

Ncl has a large IDR at its N terminus (residues 1–270) with a net charge of −40 (at pH 7). Within the Ncl N terminal IDR is a putative bipartite nuclear localization sequence [24–26]. This IDR is one of 2 low complexity regions in Ncl, the other, located at the C terminus of the protein and rich in phenylalanine and glycine (FG), was not tested in this study. Fbl also has an IDR at its N terminus (residues 1–89) with a net charge of +17. Gar1 has IDRs at both the N and C termini’s (residues 1–64 and 143–218) with net charges of +7 and +17, respectively. Both Fbl and Gar1’s IDRs are arginine and glycine rich (RG) but also contain FG sequences. The C terminal IDR of Gar1 was not altered in these experiments.

A schematic of the IDR fusions we tested can be seen in Fig 1C. We first tested if the IDRs alone, when fused to a protein not normally found in the nucleolus, would influence that proteins’ localization. In these studies, green or red fluorescent proteins were used with the IDR of Fbl (IDR-Fbl) or Ncl (IDR-Ncl). IDR-Fbl-GFP accumulated in the GC (Figs 1D and S1B). The N terminal IDR-Fbl fused to GFP was reported by others to move to the nucleolus but did not fully recapitulate the subdomain localization [6,27], and our data supports that conclusion. IDR-Ncl-GFP also localized to the nucleolus, but instead of favoring 1 compartment, it localized throughout the nucleolus (Figs 1D and S1B). We also tested the localization of Fbl and Gar1 without their N terminal disordered regions (ΔNFbl and ΔNGar1) and found that both ΔNFbl and ΔNGar1 localized to the DFC with their wild-type counterparts (Fig 1E and 1F). We note that the ΔN proteins exhibit minor (though statistically significant) diffusion into the GC, though both ΔNFbl and ΔNGar1 vastly favored the DFC (Figs S1B, 1E, and 1F). The ability of ΔNFbl to localize to the DFC is consistent with the findings of Feric and colleagues [6]. In our trials, neither Fbl nor Gar1’s localization to the DFC required their N terminal RG repeat intrinsically disordered regions. Previous experiments using Gar1’s homolog Garr1 in *C*. *elegans* differ from our result with respect to specific compartmentalization within the nucleolus. The removal of the N-terminal domain of Garr1 altered compartment-specific localization [28]. As noted above, in our system, proteins visualized augment the endogenous protein expression, whereas in the *C*. *elegans* system, they were able to analyze a homozygous mutant strain.

To further test the hypothesis that a negatively charged IDR would induce GC localization, we fused the large negatively charged IDR of Ncl to full-length Gar1 and Fbl (IDR-Ncl-Gar1 and IDR-Ncl-Fbl) as well as to Gar1 and Fbl with the coding sequences for their N terminal IDRs removed (IDR-Ncl-ΔNGar1and IDR-Ncl-ΔNFbl). These chimeras localized to the DFC (Fig 1E and 1F), indicating that the presence of an acidic disordered domain fused to Fbl or Gar1 was not sufficient to shift the predominant compartmentalization of these DFC proteins into the GC. We again note minor (though sometimes statistically significant) diffusion into the GC, though each chimera still strongly favored DFC localization (Figs 1E and 1F and S1B).

### 1,6-Hexanediol disrupts dense fibrillar component proteins, but the effects are not identical

To further examine the properties of nucleolar protein localization to subdomains of the nucleolus, we treated oocyte nuclei with 1,6-hexanediol. 1,6-hexanediol is a compound that is reported to disrupt liquid droplet-like phase separations as it disrupts weak hydrophobic interactions [29] and it has been reported to decrease Fbl hydrogel aggregation in vitro [30].

We treated freshly isolated oocyte nuclei with 10% 1,6-hexanediol and examined the effect on 2 DFC proteins, Fbl and Gar1. Within 10 min, there was visual evidence that these proteins disperse into the GC and perhaps out of the nucleolus. We collected images from multiple trials using the 10-min mark as an endpoint and found that there was a difference in the response of Fbl and Gar1 (S2A Fig) [31]. While typically, the fluorescent signals from these 2 proteins overlap completely, 1,6-hexanediol treatment diminishes the overlap as measured using Pearson’s coefficient (S2B Fig), with Gar1’s component localization being more affected than Fbl’s. 1,6-hexanediol appeared to have a greater effect on the domain localization of Gar1 than it did on its phase separation into the nucleolus as separated from the nucleoplasm. However, whether this is a direct effect on Gar1 (or Fbl) or the consequence of 1,6-hexanediol on other proteins that they interact with is not clear. It does suggest that electrostatic interactions, as would be expected from the charged intrinsically disordered regions, were not sufficient to maintain the compartmentalization profile of the proteins.

### Complex binding and nucleolar domain localization

**Fig 2:**
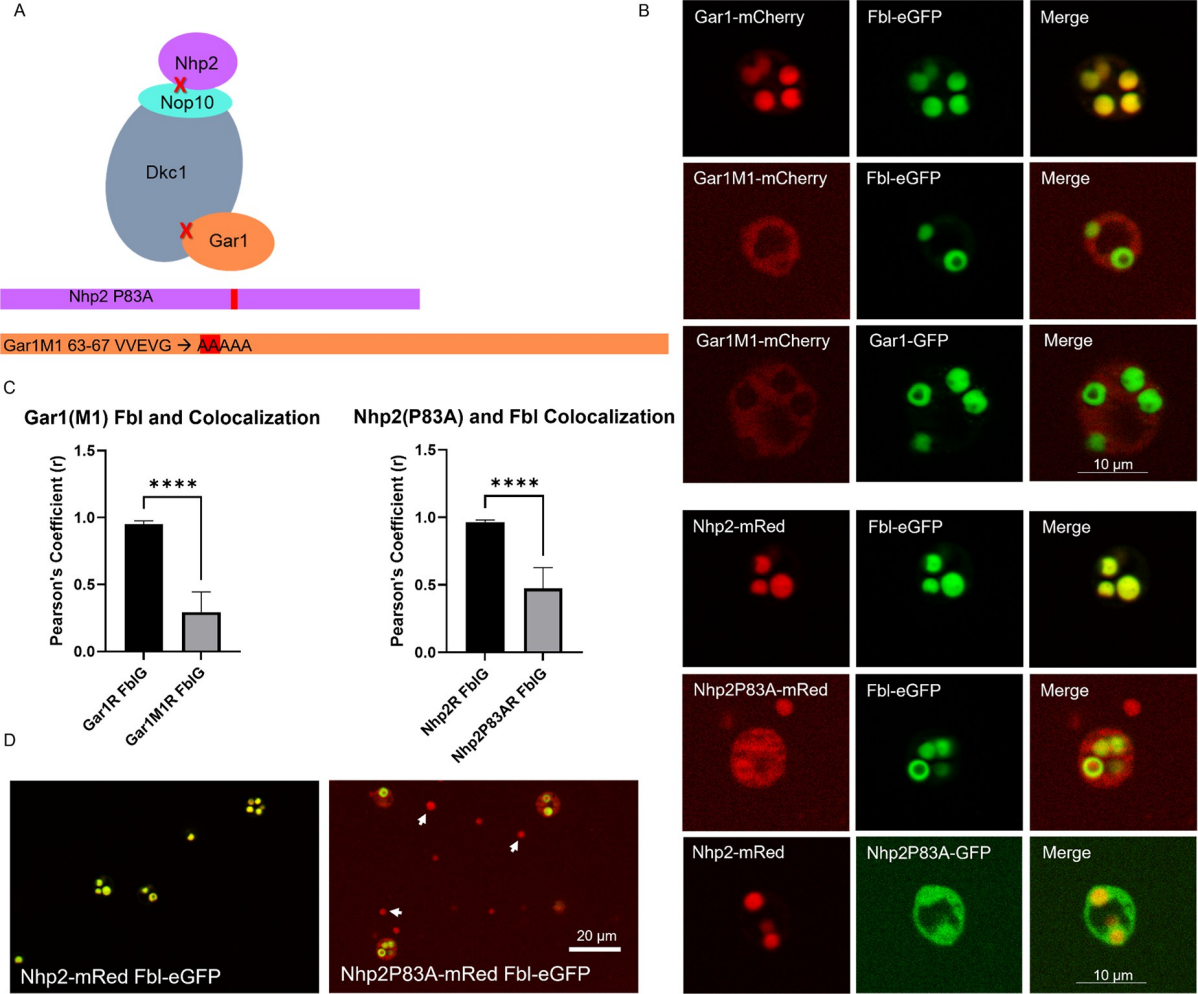
Nucleolar localization after binding disruption. **(A)** Model of the general layout of the box H/ACA complex proteins and a schematic showing the mutation sites to disrupt binding. **(B)** Representative images of nucleoli expressing wild-type Gar1-mCherry and Gar1 with a mutated binding site to DKC1 (Gar1M1-mCherry) along with the DFC marker Fbl-eGFP. Also shown are wild-type Nhp2-mRed and Nhp2 with a point mutation that disrupts its binding to Nop10 (Nhp2P83A-mRed) with DFC marker Fbl-eGFP. Isolated nuclei were incubated in OR2 for 20 min prior to imaging. Scale: 10 μm. **(C)** Quantification of the colocalization of Gar1M1-mCherry or Gar1-mCherry with Fbl-eGFP and of Nhp2-RFP or Nhp2P83A-RFP with Fbl-eGFP shown with Pearson’s coefficient. Significance was determined using a *t* test (*p* < 0.05) with *N* = 30 (Gar1) and 26 (Gar1M1) for the Gar1 set and *N* = 24 (Nhp2) and 21 (Nhp2P83A) for the Nhp2 set. These experiments were repeated with nucleoli from the oocytes of 3 different frogs and were significant each time. The underlying data can be found in S1 Data. **(D)** Images of Nhp2-mRed and Nhp2P83A-mRed showing extra-nucleolar deposits of Nhp2P83A (arrows) that were not present for the wild type. Scale: 20 μm. DFC, dense fibrillar component.

The box H/ACA complex involved in rRNA pseudouridylation contains 4 proteins: Gar1, Nhp2, Dkc1, and Nop10 (and snoRNA) (Fig 2A). We previously showed that fluorescently tagged Gar1 and Nhp2 concentrate in the DFC [5]. The binding site of human Gar1 to Dkc1 has been studied [32] and, in human, MacNeil and colleagues showed that replacing the sequence 70 VVLLG 74 with alanines significantly reduced the ability to co-immunopreciptiate Gar1 with Dkc1 [32]. An alignment of *Xenopus* Gar1 with human Gar1 suggests that residues 63–67 of *Xenopus* Gar1 are the corresponding amino acids in *Xenopus* Gar1. To test if disrupting this putative Gar1 binding site to Dkc1 would cause a change in domain localization, we mutated residues 63–67 from VVEVG to AAAAA to make Gar1M1. We found that Gar1M1 localized to the nucleolus but did not concentrate in the DFC (Fig 2B).

To determine if association with the box H/ACA complex is generally important for correct subdomain localization, we examined Nhp2. Koo and colleagues demonstrated that Nhp2’s association with the box H/ACA complex depends upon a highly conserved proline (proline 83) at the binding interface [33]. The importance of the proline, present in organisms from yeast to humans, was demonstrated both by co-immunoprecipitation experiments and by NMR as being required for its interaction with Nop10 [33]. We mutated proline 83 to alanine to make Nhp2P83A. We found that Nhp2P83A no longer accumulates primarily in the DFC, but spreads to the GC (Fig 2B).

### Introducing a binding site can induce dense fibrillar component localization

**Fig 3:**
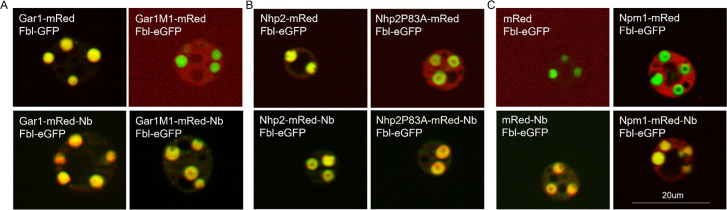
Nanobody binding can induce DFC localization. **(A)** Representative nucleoli from oocytes expressing Gar1 and Gar1M1 nucleolar localization and the same proteins fused with a GFP nanobody (Nb). These proteins were all fused with a red fluorescent protein and were co-expressed with Fbl-eGFP, a DFC marker. **(B)** Nhp2 and Nhp2P83A with and without nanobody fusion co-expressed with Fbl-eGFP. **(C)** The localization of a monomeric fluorescent protein (mRed) and Npm1 bound to the GFP nanobody, co-expressed with Fbl-eGFP. For each condition in Fig 3, 35+ nucleoli were observed from each of 3 different injection days, representative images are shown here. Scale: 20 μm. DFC, dense fibrillar component.

Site-specific changes to Gar1 and Nhp2, to disrupt binding to Dkc1 and Nop10 respectively, prevented normal localization, which indicates that the accumulation of some proteins in the DFC may depend upon their ability to stably form part of a protein complex. However, the changes in distribution seen in Fig 2 could also occur if the mutations alter the ability of the proteins to enter the DFC. To examine if the mutated proteins only lack a binding partner to stabilize their retention in the DFC, we introduced novel protein binding capacity into several proteins using GFP nanobodies. We added an anti-GFP nanobody [34] in-frame with the RFP fusions of Gar1M1 and Nh2P83A (Gar1M1-mRed-Nb and Nhp2P83A-mRed-Nb respectively) to see if DFC localization could be recovered by binding with a GFP-tagged DFC localizing protein. The addition of the GFP nanobody restored Gar1M1 and Nhp2P83A’s localization to the DFC (Fig 3A and 3B) when the DFC contained Fbl-eGFP. Additionally, adding the nanobody to proteins that do not usually localize to the DFC caused at least partial DFC localization. RFP does not accumulate in the nucleolus when expressed in *Xenopus laevis* oocytes, but when tagged with the nanobody (mRed-Nb) and co-expressed with Fbl-eGPF, it will concentrate in the DFC (Fig 3C). Npm1, a GC localizing protein, which normally appears to be nearly excluded from the DFC, begins to blend into the DFC when fused with the nanobody (Npm1-mRed-Nb) and co-expressed with Fbl-eGFP (Fig 3C). This supports our earlier conclusion that complex binding plays an important role for Nhp2 and Gar1 accumulation in, and for some proteins can be sufficient for, directed retention in the DFC.

**Fig 4:**
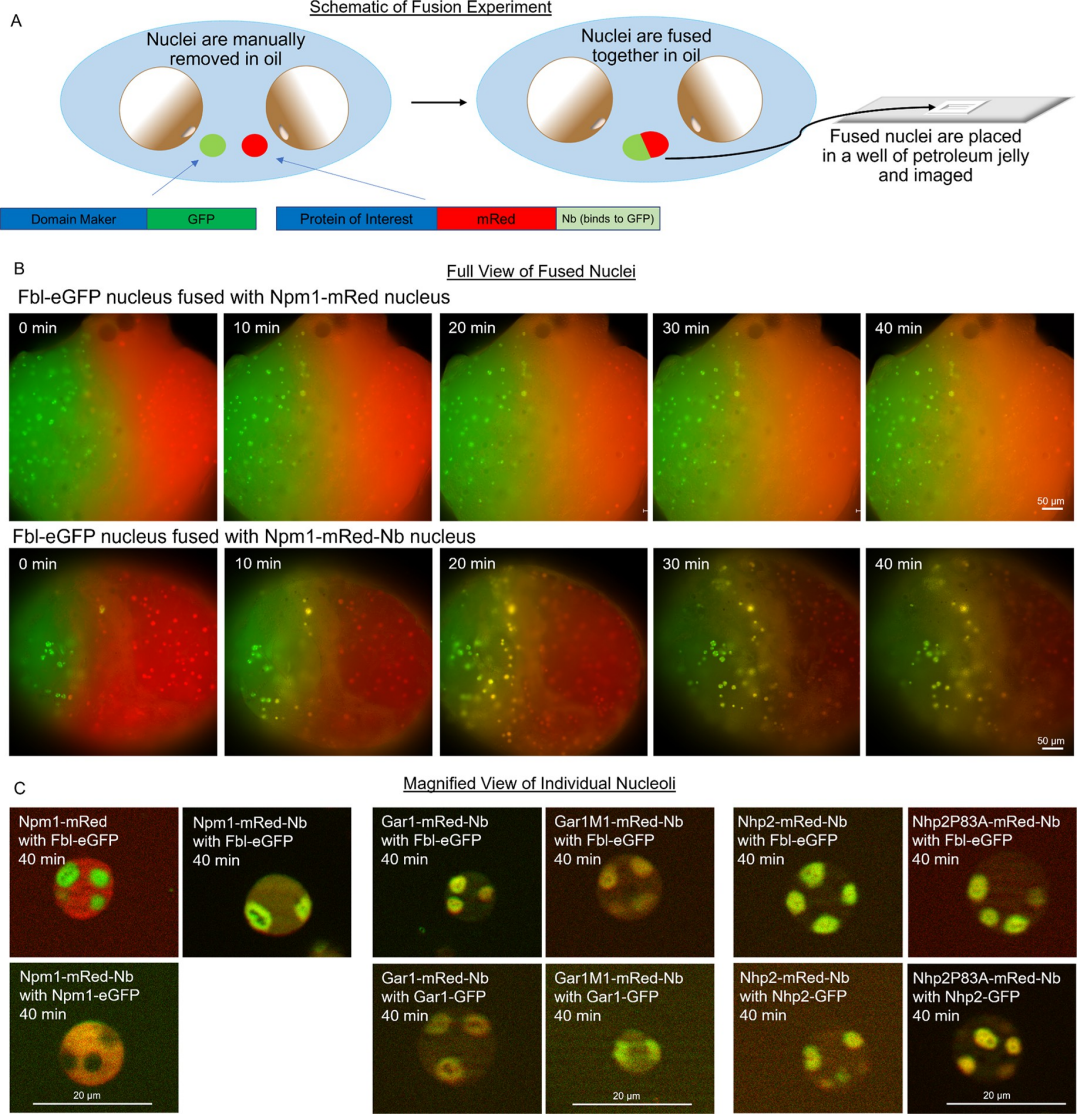
Nuclear fusion experiments. **(A)** Schematic of nuclear fusion experiments, showing nuclei being isolated in mineral oil; 2 nuclei, each expressing different fluorescently tagged proteins, being fused together; then fused nuclei being added to a microscope slide in a well of petroleum jelly for imaging. **(B)** Fluorescent images taken in 10-min intervals showing the diffusion of the fluorescently tagged proteins from 1 nucleus to the nucleus it is fused with. The top row shows a nucleus containing Fbl-eGFP fused with a nucleus containing Npm1-mRed. The second row shows a nucleus with Fbl-eGFP fused with a nucleus expressing Npm1-mRed-Nb. Scale: 50 μm. **(C)** Representative Apotome fluorescent images of nuclei found at the junction of the fused nuclei after 40 min for the proteins indicated on each image. For each condition in Fig 4, 35+ nucleoli were observed from each of 3 different injection days, representative images are shown here. Scale: 20 μm.

To determine if the nanobody-tagged proteins could bind to GFP fusion proteins within the nucleus (as opposed to binding in the cytosol and then localizing to a particular nucleolar compartment while already bound), we set up a system to allow free diffusion across 2 nuclei as previously carried out by Hayes and colleagues [7]. Nuclei from oocytes expressing a single fluorescent fusion protein of interest were isolated in mineral oil to limit the diffusion of proteins out of the nucleus (Fig 4A). Isolating nuclei in mineral oil retains physiological nuclear content (nuclear chaperones, approximate ATP and salt concentration, other small molecules, and potential co-aggregates). As controls, we show the diffusion of the expressed proteins Npm1-mRed or Fbl-eGFP between fused nuclei compared to nuclei isolated with the anti-GFP nanobody fusion Npm1-mRed-Nb co-expressed with Fbl-eGFP (Fig 4B and 4C). At the beginning of the fusion process, each nucleus has a single fluorescent protein. Npm1-mRed and Npm1-mRed-Nb occupy the GC in nuclei glowing red, Fbl-eGFP is in the DFC in nuclei glowing green (Fig 4B). The images also show that, although there is an enhanced accumulation of these proteins in the nucleolus, there remains a significant pool in the nucleoplasm. Over time, nucleoli from each of the nuclei accumulate the fluorescent protein from the nucleus their fusion partner. In the case of Npm1-mRed and Fbl-eGFP, the stable distribution is with Npm1 in the granular compartment and Fbl-eGFP in the DFC (Fig 4C). In contrast, the experiments using Npm1-mRed-Nb show distribution of the proteins across both the GC and the DFC (Fig 4C). These experiments show that proteins tagged with the nanobody can diffuse into and accumulate with GFP-tagged proteins in the nucleolus within the nucleus and do not require cytosolic binding (Fig 4B and 4C). We show this is true for both Gar1M1 and Nhp2P83A (Fig 4C).

## Discussion

The compartments of the nucleoli found in *Xenopus laevis* oocytes are visually distinct whether assayed using electron microscopy or through tracking compartment-specific proteins as described by Feric and colleagues and others including from our lab [2,5,7]. The components of each compartment are consistent with a role in organizing the progressive assembly of ribosomes, from rRNA transcription to rRNA modification then to rRNA processing and ribosome assembly. Handwerger and colleagues estimated that the concentration of protein in the nucleoli is almost double that found in the nucleoplasm [35], and proteins in the nucleolus are generally thought to exchange with their non-nucleolar pools in the nucleoplasm [36]. Free diffusion between the nucleoli and nucleoplasm is affected by particle size, reminiscent of diffusion limits imposed by nuclear pores and P-granules in other systems [35]. The rate of diffusion into and out of nucleoli is affected by particle size, but even particles of 1,000 kDa appear to be able to enter and leave nucleoli. Diffusion rates were reported to be compartment-specific and slower in the DFC and FC than in the GC [35].

Our understanding of why proteins selectively join 1 phase-separated domain over another is evolving. In cytosolic stress granules, the acidic disordered region of the protein G3BP is necessary for the formation of multi-domain stress granules, as G3BP aggregation is destabilized by the charge repulsion between the acidic IDR and the negative backbone in RNA [37]. Charge differences in the IDRs of synaptophysin and synapsin provide electrostatic support for the formation of a protein condensate that organizes a pool of synaptic vesicles [39]. These studies, along with our finding that acidic IDRs are enriched in the GC led us to investigate if the charges of the IDRs play a driving role in nucleolar domain localization. Our initial hypothesis was that the presence of a negatively charged IDR would alter DFC localization and drive a protein into the GC. In this analysis, we used the large (31 kDa), negatively charged (pI 4.42) IDR from Ncl. The addition of this IDR to Gar1 or Fbl did not prevent their localization to the DFC. Placing the positively charged IDR of the DFC localizing protein Fbl (IDR-Fbl: approximately 10 KDa, pI 12.34) onto GFP did not selectively compartmentalize GFP to the DFC. While the charges of a protein’s IDR may play some role in nucleolar localization/function [38], they do not appear to be sufficient to drive compartment localization for the proteins we have tested. As shown in Fig 1, Ncl has somewhat variable localization in our system. Ncl was included in the proteome of proteins associated with the new fourth nucleolar domain, the PDFC. These authors conclude that Ncl is a high-mobility component of the PDFC and our observations of the full-length protein in both the GC and occasionally the DFC may reflect the ease of entry from the PDFC into these compartments [2,3].

Whether IDRs are necessary and sufficient for phase separation may depend upon the protein and its cellular compartment. For Fbl and Gar1, the presence of the N-terminal IDR was not required for nucleolar or DFC-specific accumulation, as ΔNFbl and ΔNGar1 both localize to the DFC. It is important to note that we were careful to leave the Dkc1 binding site on Gar1 (which is found at the junction between the N terminal IDR and the ordered region of the protein) intact when making ΔNGar1.These observations on Fbl are consistent with the findings of Feric and colleagues in a similar experiment who used *Xenopus* oocytes in their studies [6], but contrast somewhat with the studies in *C*. *elegans* that found that removal of either the N or the C terminal altered the sub-nucleolar distribution of Gar1 [28]. In our study, the truncated protein, and all the proteins being studied here, were expressed by translation of injected mRNA, and joined the pool of wild-type protein. In the *C*. *elegans* experiments, Garr1 mutants were generated in the Garr1 genomic site to assure similar levels of expression to that found in wild type.

Caution is merited when interpreting the effects of 1,6-hexanediol, especially when used to examine multicomponent biological systems rather than purified proteins [31,39]. However, both Fbl and Gar1 were affected when nuclei were treated with 1,6-hexanediol, though the effects were not identical. We found it particularly interesting that Gar1 remained in the GC after 1,6-hexanediol treatment, while Fbl dispersal favored transiting the GC and moving into the nucleoplasm. 1,6-hexanediol interferes with weak hydrophobic interactions. The hydrophobicity of Fbl is slightly more than Gar1, both comparing the full protein and just the IDRs (Fbl: 36.94%, 16.83%. Gar1: 26.15%, 9.65% [values were found using Kyte and Doolittle hydrophobicity analysis [40]]). Whether these differences in hydrophobicity account for the different phenotypes of Fbl and Gar1 after treatment is unclear. The N-terminal IDR for Fbl also contains more FG repeats than Gar1. FG repeats are thought to coordinate scaffold-like interactions mediated via the hydrophobic interaction of phenylalanine and this may also be a factor in our observations. However, whether the changes in localization are direct or indirect effects of 1,6-hexanediol are hard to determine. In general, we would suggest that 1,6-hexanediol treatment of the mixed condensates found in biological systems provides useful but modest information.

The box H/ACA complex is responsible for the pseudouridine modification of rRNA and includes 4 proteins found in the mature complex [41]. Dkc1 has a direct interaction with Nop10 and the Dkc1/Nop10 complex mediates the addition of Nhp2, while Gar1 had a direct but relatively weak interaction with Dkc1 [32,41]. Initial assembly of the complex includes DKC1, Nop10, and Nhp2 [41,42]. A fifth factor, Naf1 has been identified as critical for H/ACA assembly, as it facilitates Dkc1, Nop10, and Nhp2 interaction as well as nuclear accumulation and is replaced by Gar1 in the mature complex [43,44].

Although neither the loss of Gar1’s endogenous N-term IDR nor the addition of the acidic IDR from Ncl prevented Gar1 from predominately localizing to the DFC, mutating the putative binding site between Gar1 and Dkc1 led to significantly lower levels of Gar1 in the DFC, even though the mutant Gar1 protein is still present in the nucleolus. This indicates Gar1 may represent a class of nucleolar proteins that can enter the nucleolus, diffuse into multiple compartments but only accumulate in the DFC by binding to something that is already there. Like Gar1, Nhp2 localization changed after disruption of a conserved proline residue previously shown to be important for interaction with Nop10 [33]. This provides a second example where domain concentration depends on the ability to bind to a protein complex. We note that our attempts to confirm H/ACA complex assembly by co-immunoprecipitation reactions using material from *Xenopus* oocytes proved problematic. Extract preparation that left the nucleolar H/ACA complex intact left many nucleolar proteins precipitable even with low-speed centrifugation. Our conclusion that the mutations examined disrupt binding of Gar1 or Nhp2 to the protein complex assumes the *Xenopus* complex behaves like those described in human [33]. Interestingly, while the mutant Nhp2P83A appeared to phase separate into the nucleolus, it also accumulated in extra-nucleolar spots (Fig 2D), something which we have never seen for wild-type Nhp2. This phenotype may suggest that without retention in the DFC, there is a limit to how much Nhp2 can occupy the nucleolus, though it still favors phase separation.

The mutated forms of Gar1 and Nhp2 can enter the DFC. The fusion of an in-frame GFP nanobody to the mutants and co-expression of Fbl-eGFP to provide a novel binding combination restores Gar1M1 and Nhp2P83A’s DFC accumulation. This was true whether the oocytes were simultaneously injected with RNA encoding a nanobody-tagged protein along with a GFP-tagged protein, or by fusing nuclei in oil that separately contained either nanobody or GFP-tagged protein. This shows that nanobody-tagged Gar1M1 and Nhp2P83A can concentrate in the dense fibrillar component if a binding partner is present. These studies also indicate that proteins that are not normally part of the stable pool of proteins in the nucleolus, like mRed, can accumulate in this mixed condensate if provided with an appropriate binding partner.

The experimental expression of fluorescent protein fusions of the H/ACA complex proteins Gar1 and Nhp2 showed that the pools of these proteins within nucleoli are not static. The fluorescent fusion proteins become part of the pool of endogenous Gar1 and Nhp2 and join existing nucleoli. We note here our inability to show that fluorescent protein fusions of Dkc1 or Nop10, the other 2 members of the complex, mimic this behavior. Although the injection of mRNA encoding fluorescent fusions of Dkc1 and Nop10 generate proteins that move to the nucleus, any accumulation in the nucleoli was below our detection ability. This was not the case when the Dkc1 mRNA was injected into embryos. The box H/ACA complex is typically represented as consisting of stoichiometric levels of Dkc1, Nop10, Nhp2, and Gar1, and concentration estimates of these 4 proteins in *Xenopus* eggs are within 20% of each other [45]. This observation suggests that modeling RNA modification complexes like the box H/ACA complex may need to include how the nucleolus establishes a limit on the total number of complexes that can be accommodated. The model must also allow for some proteins in a condensate-based complex to be in rapid exchange, while others are not.

What anchors the DFC? A scaffold-client model of phase separation has previously been proposed, where one protein phase separates and other proteins are able to join the phase because they can interact with the scaffold [46]. This model has been used to describe the addition of soluble proteins to phase-separated condensates, where the soluble proteins are not able to phase-separate alone. With the multi-compartment nucleolus, a scaffold/client separation could explain the separation between the DFC and the GC, where already phase-separating proteins can accumulate in the DFC only when they can bind to an existing scaffold. If the nucleolus follows the scaffold-client model of phase separation, then both Gar1 and Nhp2 appear to be client proteins. A potential scaffold protein for the H/ACA complex might be Dkc1. One could also imagine that a fraction of Fbl if bound to a co-aggregate like rRNA might serve as a scaffold for the box C/D complex, while part of the Fbl pool is in more dynamic exchange. A scaffold role for Fbl or Dkc1 or some other nucleolar component awaits experimental verification.

## Materials and methods

### Ethics statement

All protocols were approved by the University of Iowa Office of Animal Resources and Institutional Animal Care and Use Committee (Protocol # 0121363).

### Oocyte removal and preparation

Oocytes were surgically removed from *Xenopus laevis* and prepared for the assays here as described previously [5]. Briefly, frogs were anesthetized in a 0.8% to 0.1% tricaine bath for 15 min immediately prior to aseptic removal of ovary. For some trials, follicle cells were manually removed from oocytes, and oocytes were injected with mRNA a few hours later. Other trials used oocytes that were defolliculated using Collagenase (Worthington, type 1). Any residual follicle cells were removed manually with watchmaker forceps. Collagenase-treated oocytes recovered at 13°C overnight in Oocyte Culture Medium before further treatment [47]. *Xenopus laevis* were purchased from Xenopus-1 (Dexter Michigan, United States of America).

### Construction of protein fusion cDNA

cDNA encoding the fluorescent fusions of Npm1 and Fbl tagged with either mRed or eGFP were a gift from the Cliff Brangwynne laboratory (Princeton University, New Jersey). cDNA of the other wild-type nucleolar proteins were purchased from TransOmic Technologies and fluorescent fusions also included GFP (mGFP5 [48]) and mCherry [49]. These were made as described previously [5]. All constructs were put into the RN3P plasmid backbone (a gift from John Gurdon, Cambridge University, United Kingdom) and the sequences encoding the IDRs were assembled using NEB’s Q5 PCR and Gibson’s Assembly (New England Biolabs, E5510S) as described previously [5]. It is important to note that while the N terminal IDR of Gar1 was predicted to be residues 1–64, Gar1’s binding site with DKC1 starts at residue 63. To preserve this binding site, ΔNGar1 was made by deleting residues 1–60 of Gar1. The IDR of Ncl is residues 1–270, and the IDR of Fbl is residues 1–89.

Gar1M1 was made using the template Gar1-mCherry and Gar1-GFP [5]. Residues 63–67 were changed from VVEVG to AAAAA using site-directed mutagenesis (NEB’s KLD reaction mix, M0554). Nhp2P83A was made by changing proline 83 to alanine in Nhp2 in the same manner. After the KLD reaction or the Gibson Assembly, plasmids were transformed in NEB Stable Competent *E*. *coli* cells (for Gar1, and Ncl containing fusions, C3040H) or NEB’s 5 alpha *E*. *coli* cells (for all other fusions, C2987H). For nanobody experiments, cDNA encoding the “enhancer” nanobody from Kubala and colleagues was purchased from Addgene [34]. The enhancer was amplified using PCR and added to the C terminal ends of previously made RFP-tagged protein clones using Gibson Assembly. See S2 Table for a list of primers and fluorescent proteins for each fusion. All constructs were verified by sequencing (University of Iowa Genomics Core).

### mRNA in vitro synthesis and injection into oocytes

NEB’s Q5 PCR was used to create a linear template for in vitro transcription using the primers m13F and m13R. mRNA was made using mMessage mMachine’s T3 in vitro transcription kit using the recommended protocol (Ambion). Production of mRNA was verified by analysis using agarose gel electrophoresis and concentration was determined using a Nanodrop spectrophotometer (Thermo Scientific). Aliquots were stored at −80°C until use, and 10 nL of mRNA at a concentration of 100 ng/μL was injected directly into stage V-VI oocytes using a Singer MK-1 and a glass needle and an Inject+Matic injector (Geneva, Switzerland) as described previously [5]. The expression of fluorescent fusions was verified using SDS PAGE analysis and immunoblots using GFP (Living Colors, JL-8) and RFP (Chromotek, 6G6) antibodies. To estimate the amount of protein added to the endogenous pool, we compared the expression of Ncl-GFP with endogenous Ncl using the monoclonal antibody b6-6E7 developed by C. Dreyer at Max-Plank-Institut fure Entwicklungsbiologie obtained from the Developmental Studies Hybridoma Center, created by the NICHD of the NIH and maintained at the University of Iowa, Department of Biology, Iowa City, Iowa 52242. These trials indicate that under the conditions described the endogenous pool of proteins is increased between 5% and 20%.

### Apotome fluorescent microscopy

Nuclei were isolated manually in Oocyte Ringers Solution (OR2, 82.5 mM NaCl, 2.5 mM KCl, 1 mM CaCl_2_, 1 mM MgCl_2_, 1 mM Na_2_HPO_4_, 5 mM HEPES, and NaOH to pH 7.8) under a dissection microscope. Isolated nuclei were placed on a microscope slide in a well of petroleum jelly and covered with a coverslip and nucleoli that drifted to the bottom of the nucleus were imaged using an AxioPlan or ApoTome fluorescent microscope running Axio Vision software (RRID:SCR_002677) and an AxioCam Mrm or AxioCam MrC5 camera (Zeiss) as described previously [5]. When indicated (as is shown in Fig 2) nuclei remained in a dish of OR2 for 20 min prior to imaging to allow soluble proteins to diffuse. This was done when the background glow of soluble proteins (injected fluorescent fusions) made it challenging to clearly image the nucleoli. The analysis of images taken after 20-min diffusion and those imaged immediately both produced the significant difference in WT versus mutated shown in Fig 2 (S1 Data). Some frogs contained oocytes that had nucleoli with minimal green background signal. When needed, datasets were normalized to controls not injected with green fluorescent proteins.

### 1,6-Hexanediol treatment

Nuclei were isolated manually in OR2 and immediately moved (while being careful to keep the nuclear envelope intact) into a dish containing a solution of 10% 1,6-hexanediol solution (Sigma-Aldrich) dissolved in OR2. Nuclei were incubated at room temperature in 1,6-hexanediol for 10 min. Nuclei were then placed on a microscope slide in a well of petroleum jelly and covered with a coverslip and imaged immediately.

### Co-localization image analysis

Co-localization image analysis using Pearson’s coefficient was used to assess the overlap of Gar1M1 and Nhp2P83A with a DFC marker (Fbl) shown in Fig 2 and to assess Gar1/Fbl overlap after 1,6-Hexanediol treatment shown in S2 Fig. For this, images were cropped around individual nuclei, and then channels were split in ImageJ. The JACoP plugin in ImageJ was then used to assess the Pearson’s coefficient group between control and treatment groups. All graphs were made, and all statistical analysis was done using GraphPad Prism (*p* < 0.05). These experiments were repeated at least 3 times with oocytes from 3 different frogs and with the fluorescent tags switched (Gar1 tagged with a red fluorescent protein and Fbl with a green fluorescent protein and vice versa) and found significant each time. See S1 Data and S3 Data. Co-localization using Pearson’s coefficient was also used to assess Ncl-GFP and Fbl-RFP overlap from different days as shown in S1 Fig (S2 Data).

The variation of each intrinsically disordered region chimera’s localization within the nucleolus was quantified using the coefficient of variation (shown in S1 Fig) as previously described by Spaulding and colleagues [28]. Briefly, images were split into fluorescent channels and for each channel individual nucleoli were selected and *getRawStatistics(N*, *mean*, *min*, *max*, *std);* and *print(std/mean);* commands were used to determine the coefficient of variation for each fluorescent marker. A summary of this data can be found in S1 Fig and in S2 Data.

### Nuclear fusion experiments

Oocytes were injected with mRNA encoding either a red fluorescently tagged protein or a green fluorescently tagged protein. After allowing 24 to 72 h for protein expression, nuclei were manually isolated in a dish of light mineral oil (Sigma-Aldrich 33079). A small tear was made in the nuclear envelopes of 2 nuclei (1 containing a GFP-tagged protein and 1 containing an RFP-tagged protein) nuclei were fused together at the point of the tear. The fused nuclei were then carefully placed on a microscope slide in a well of petroleum jelly and covered with a coverslip. The fused nuclei were imaged every 10 min for 40 min using fluorescent microscopy. After 40 min, nucleoli were imaged at a higher magnification using the ApoTome fluorescent microscope.

## Supporting information

S1 Fig Statistical analysis for Fig 1. **(A)** Statistical analysis using Pearson’s Coefficient to assess the overlap of fluorescently tagged Ncl co-expressed with fluorescently tagged Fbl on 4 different days using one-way ANOVA multiple comparisons test, *p* < 0.05. *N* = 15, 58, 52, and 24 for days 1–4 respectively. **(B)** Table showing the results of the statistical analysis comparing the coefficients of variations of the conditions indicated in the left column. Replicates “1, 2, and 3” indicate different frogs/injection days for each condition. Values from columns are not necessarily from the same day (data should be read horizontally and not vertically). *P* < 0.05, *N* values between 15–62. The underlying data can be found in S2 Data.
(TIF)

S2 Fig Response of Fbl and Gar1 to 1,6-hexanediol treatment. **(A)** Representative images of nucleoli expressing Gar1-GFP and Fbl-mRed from nuclei that were isolated in OR2 and soaked in 10% 1,6-hexanediol for 10 min. **(B)** Analysis of co-localization using Pearson’s coefficient between Gar1 and Fbl with and without hexanediol (*p* < 0.05) *N* = 35 (control) and 33 (hexanediol). This experiment was repeated with nucleoli from the oocytes of 3 different frogs and with the fluorescent tags switched. The underlying data can be found in S3 Data.
(TIF)

S1 Table Disorder prediction and nucleolar localization. Column 1: Protein name. Column 2: Pondr-FIT prediction of intrinsically disordered regions(s) (IDR), with residue number on the X axis and predicted disorder (>0.5) on the Y axis. Column 3: Primary sequence, with IDRs (as predicted by Pondr-FIT, Xue and colleagues [20]) are highlighted in yellow and putative binding sites of Gar1 and Nhp2 that were mutated in this article are shown in red. Column 4: Nucleolar localization of the proteins, largely based on Lavering and colleagues (2022) [5].
(DOCX)

S2 Table Cloning primers. This table shows the primers used to make the constructs shown in the paper and the fluorescent protein in each construct. They were made using Gibson Assembly or Q5 Site-Directed Mutagenesis as indicated in subheadings. *These were made only by amplifying the region of interest using the given primers using Q5 PCR and using the amplified region as a template for mRNA transcription.
(DOCX)

S1 Data Quantification of the colocalization of Gar1M1-mCherry or Gar1-mCherry with Fbl-eGFP and of Nhp2-RFP or Nhp2P83A-RFP with Fbl-eGFP from Fig 2 shown with Pearson’s coefficient. Significance was determined using a *t* test (*p* < 0.05) with *N* = 30 (Gar1) and 26 (Gar1M1) for the Gar1 set and *N* = 24 (Nhp2) and 21 (Nhp2P83A) for the Nhp2 set. These experiments were repeated with nucleoli from the oocytes of 3 different frogs and were significant each time (Supplementary data file: S1_Data.xlsx).
(XLSX)

S2 Data Statistical analysis using Pearson’s Coefficient to assess the overlap of fluorescently tagged Ncl co-expressed with fluorescently tagged Fbl on 4 different days using one-way ANOVA multiple comparisons test, *p* < 0.05. *N* = 15, 58, 52, and 24 for days 1–4, respectively (Supplementary data files for Figs 1D, 1F, S1A and S1B can be found in: S2_Data.xlsx).
(XLSX)

S3 Data Comparing the colocalization (Pearson’s Coefficient) of Fbl/Gar1 with and without 1,6-Hexanediol Treatment as shown in S2 Fig. Analysis of co-localization using Pearson’s coefficient between Gar1 and Fbl with and without hexanediol (*p* < 0.05) *N* = 35 (control) and 33 (hexanediol). This experiment was repeated with nucleoli from the oocytes of 3 different frogs and with the fluorescent tags switched (Supplementary data file: S3_Data.xlsx).
(XLSX)

## Abbreviations

DFC: dense fibrillar component
FC: fibrillar center
GC: granular component
IDR: intrinsically disordered region
PDDF: periphery of the dense fibrillar component
