# Supplementary figures and images for: Intrinsically disordered regions are not sufficient to direct the compartmental localization of nucleolar proteins in the nucleus
Source: PLoS Biol. 2023 Nov 9;21(11):e3002378. doi: 10.1371/journal.pbio.3002378 (PMC10662738; doi:10.1371/journal.pbio.3002378)

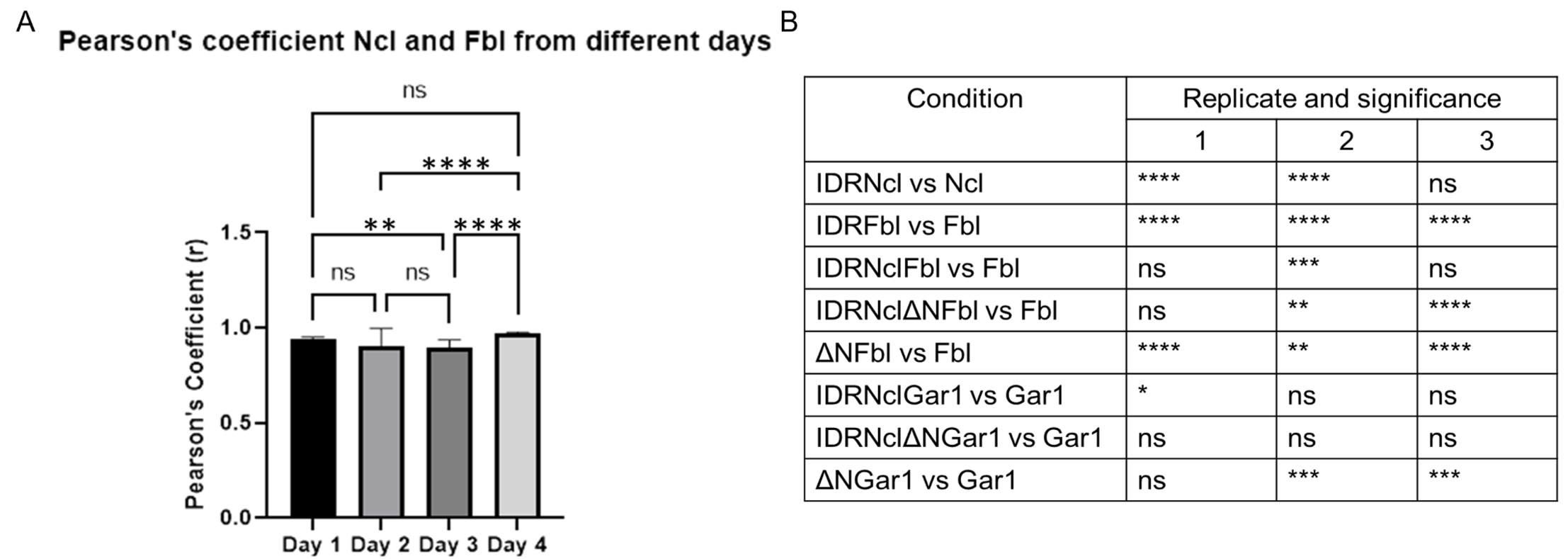

Supplement: S1 Fig — (A) Statistical analysis using Pearson’s Coefficient to assess the overlap of fluorescently tagged Ncl co-expressed with fluorescently tagged Fbl on 4 different days using one-way ANOVA multiple comparisons test, p < 0.05. N = 15, 58, 52, and 24 for days 1–4 respectively. (B) Table showing the results of the statistical analysis comparing the coefficients of variations of the conditions indicated in the left column. Replicates “1, 2, and 3” indicate different frogs/injection days for each condition. Values from columns are not necessarily from the same day (data should be read horizontally and not vertically). P < 0.05, N values between 15–62. The underlying data can be found in S2 Data. (TIF) [file pbio.3002378.s001.tif]

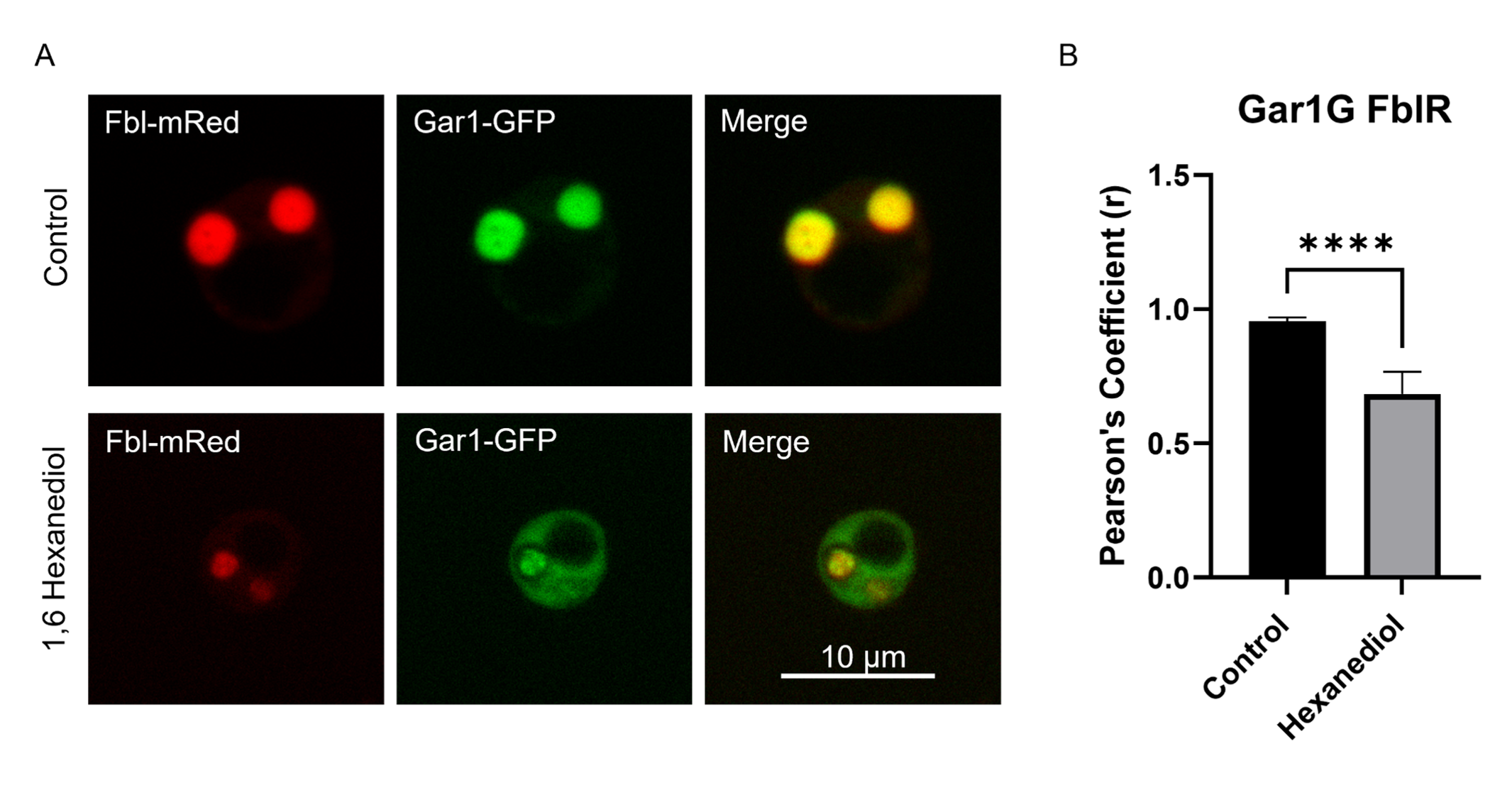

Supplement: S2 Fig — (A) Representative images of nucleoli expressing Gar1-GFP and Fbl-mRed from nuclei that were isolated in OR2 and soaked in 10% 1,6-hexanediol for 10 min. (B) Analysis of co-localization using Pearson’s coefficient between Gar1 and Fbl with and without hexanediol (p < 0.05) N = 35 (control) and 33 (hexanediol). This experiment was repeated with nucleoli from the oocytes of 3 different frogs and with the fluorescent tags switched. The underlying data can be found in S3 Data. (TIF) [file pbio.3002378.s002.tif]
