# Supplementary material for: Intrinsically disordered regions are not sufficient to direct the compartmental localization of nucleolar proteins in the nucleus
Source: PLoS Biol. 2023 Nov 9;21(11):e3002378. doi: 10.1371/journal.pbio.3002378 (PMC10662738; doi:10.1371/journal.pbio.3002378)
Supplement: S1 Table — Column 1: Protein name. Column 2: Pondr-FIT prediction of intrinsically disordered regions(s) (IDR), with residue number on the X axis and predicted disorder (>0.5) on the Y axis. Column 3: Primary sequence, with IDRs (as predicted by Pondr-FIT, Xue and colleagues [20]) are highlighted in yellow and putative binding sites of Gar1 and Nhp2 that were mutated in this article are shown in red. Column 4: Nucleolar localization of the proteins, largely based on Lavering and colleagues (2022) [5]. (DOCX) [file pbio.3002378.s003.docx]

Supplementary Table 1

| Name  (NCBI ID) | Pondr-FIT predictions of disorder | Primary sequence | Nucleolar domain |
| --- | --- | --- | --- |
| Fbl  (NP_989101.1) | 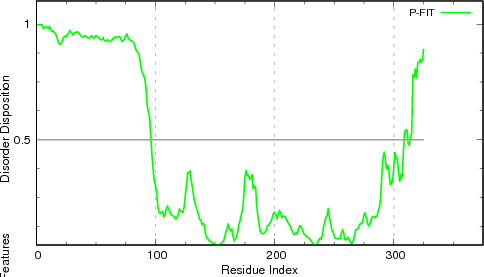 | MRPGFSPRGGRGGYGDRGGFGDRGGGRGRGGFRGRGGGGDRGGFGGRGGFGGRGGFGDRGGFRGGFKSPGRGGPRGGRGGRGGFGAGRKVIVEPHRHEGIFICRGKEDALVTKNLVPGESVYGEKRISVEDGEVKTEYRAWNPFRSKIAAAILGGVDQIHIKPGAKVLYLGAASGTTVSHVSDVVGPEGLVYAVEFSHRSGRDLINVAKKRTNIIPVIEDARHPHKYRMLVGMVDVVFADVAQPDQTRIVALNAHNFLKNGGHFVISIKANCIDSTAAPEAVFAAEVKKMQQENMKPQEQLTLEPYERDHAVVVGIYRPPAKQKK | Dense fibrillar component |
| Gar1  (NP_001011252.1) | 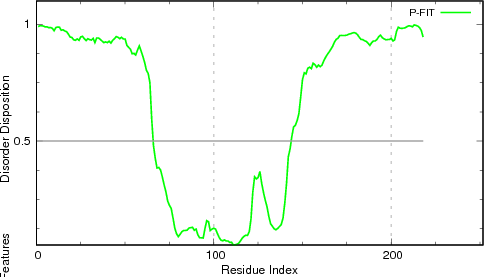 | MSFRGRGGFNRGGGGGRGGGGFGGRGGGRGGYGQGGGRGGFGRGGGRGGFNRGGYDQGPPESVVEVGEFMHPCEDDVVCKCITQENRVPYFNAPIYLENKEQIGKVDEIFGQLRDFYFSIKLSEMKASSFKKLQKFYIDPAKLLPLQRFLPRPPGEKGPPRGGGRGGGRGGGRGRGGGRGGGGGFRGGRGGGFGGGGGFRGSRGGGFRGGRGFRGGR | Dense fibrillar component |
| Nhp2 (Q6NTV9.1) | 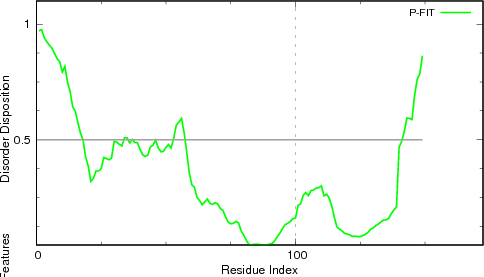 | MTKVKKEECEEVPETPSKSYDELLSYLNPVAKPLAGRKLTKKLYKCVKKAIKQKNIRRGVKEVQKFINKGEKGIVVMAGDTLPIEVYCHIPVMCEDRGIPYSYVPSKSDLGAAAGSKRPTCVILIKPHEDYQEAYDECLEDVQALPLPY | Dense fibrillar component |
| Nop56  (AAI06207.1) | 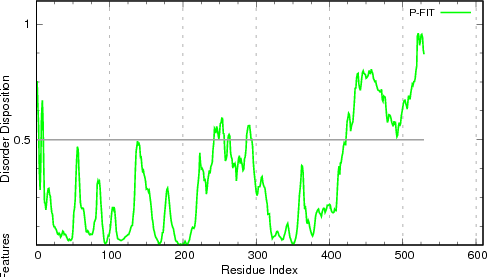 | MVLLHVLFEHAAGYALFAVKEVEEVGLLIPQVEETLLNVGKFNNIVKLAAFSPFKSAQSALENVNAISEGVLHEDLKLLLETHMPAKKKKALLGVADAKIGAAIQEELKIPCQTGGAVVEILRGIRLHFHSLVKGLTAQSASKAQLGLGHSYSRAKVKFNVNRVDNMIIQSISLLDQLDKDINTFSMRVREWYGYHFPELIKIVSDNYTYCRMAKFIGNRKELSEEKLEEMEEIVMDSAKAQAVLDASRSSMGMDISPIDLINIESFSSRVISLSEYRKELQEYLRSKMSQVAPSLSALIGEVVGARLISHAGSLTNLAKYPASTVQILGAEKALFRALKTRGNTPKYGLIFHSTFIGRAAMKNKGRISRYLANKCTIASRIDCFSEIPTSVFGDKLREQVEERLAFYETGEVPRKNLDVMKEAQQEATEVVSEVKRKLEKKEKKRKKREKRQLEALAAEEPSQKKTKENGEEDEEPKKKKKKRHSEAEVSENGMEEETSSKKKKKNTEPEEAPQKPKKKKKSKVETES | Primarily dense fibrillar component, with minor granular component localization |
| Npm1  (NP_001079507.1) | 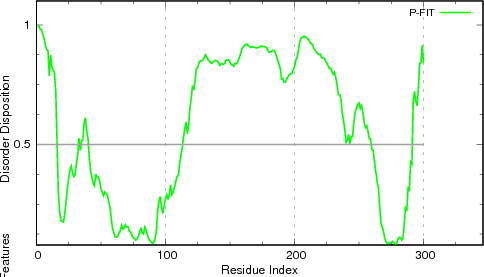 | MEDSMDMDNIAPLRPQNFLFGCELKADKKEYSFKVEDDENEHQLSLRTVSLGASAKDELHVVEAEGINYEGKTIKIALASLKPSVQPTVSLGGFEITPPVILRLKSGSGPVYVSGQHLVALEDLESSDDEDEEHEPSPKNAKRIAPDSASKVPRKKTRLEEEEEDSDEDDDDDDEDDDDEDDDEEEEETPVKKTDSTKSKAAQKLNHNGKASALSTTQKTPKTPEQKGKQDTKPQTPKTPKTPLSSEEIKAKMQTYLEKGNVLPKVEVKFANYVKNCFRTENQKVIEDLWKWRQSLKDGK | Granular component |
| Ncl (AAI70089.1) | 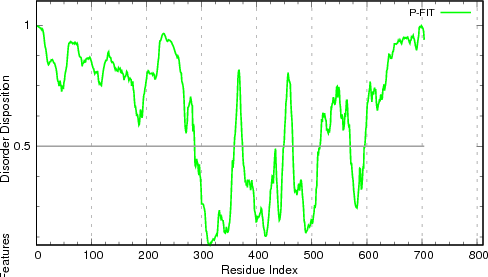 | MVKLAKGAKTQAKPKKAAPPPPKDMDDSDEEEDSEDESSEEEMEVPVKKTPAKKAATPAKATPGKAATPAKATVTPGKKGATPGAKNGKQAKEQESEEEEDDSDEEDQKPIKKPIAKKAVAKKEESEEDDDDEDEDESEEEKPVAKRPLAKNPPVKVTPAKKSTGKKQESDDEEDESEEEKPVAKKPPAKKPAGKKQESEDEESDEDEEEPMEVASAQKGKKTAPAKAEDDDDDDDDDDDDDEDDEEDDDDEQQGPAKRKKEMPKNNVPEAKKTKTDTASEGFSIFIGNLNSTLDFDELKDALREFFSKKNLTIQDVRIGGTKKFGYVDFSSEEEVEKALKLSGKKILGLEVKIEKAVAFDKNKNAENKKERDSRTLFVKNIPYSTSAEELQEIFENAKDIRIPTGNDGSNKGIAYVEFSTEAEANKALEEKQGAEIEGRSLFVDFTGEKSQNSGGRRGPAGDSKVLVVNNLSYSATEDSLREVFEKATSIRIPQNQGRAKGFAFVEFSSMEDAKEAMDSCNNTEVEGRSIRLEFSQGGGPQGGGRGGSVQSKTLFVRGLSEDTTEETLKEAFDGSINARIVTDRDTGASKGFGFVDFSSSEDAKAAREAMEDGEIDGNKVTLDFAKPKGDSQRGGRGGFGGGRGGFGGRGGGRGGFGGRGGGRGGFGGRGGGGGRGRGGFGGRGGGGFGGGNQGQGKKIRFDD | Primarily granular component, with some localization to the dense fibrillar component. There is some variation from frog to frog, with the amount of Ncl in the dense fibrillar component |
| Gtpbp4 (NP_001104215.1) | 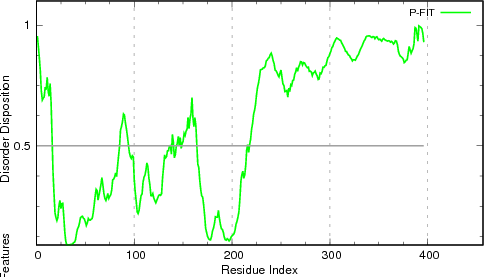 | MQAITALAHLRSAILYVMDISEQCGQSIEQQLELFSNIRPLFSNKPLIIVVNKCDIKRVSELPEEQQKIFQDLENEGLTVIETSTLTEEGVMTVKTEACDRLLVHRVENKMKGNKVNDVLNRLHLAIPAKRDGKERLPFIPEGALERKKRMVTDAPKKRLERDIEMELGDDYILDLQKYWDLMNPTEKQDIIPEIWQGHNIADYIDPEIMKKLEELEKEEELREGAGEYDSDPESEDEEMVEIRELAQQIREKKKLKFLASKEKDIHGPRLPRTAKKVQRKSLEKEMSSLGLDMTEKDETHYATQARSRSVQRKRKREESEPPATRSRSRSVSKTPRDQSGMRDVKMVKKAKKIMKNSQKKNNRLGKKGEGDRHVFDLKPKHLFSGKRKSGKTDSR | Granular component |
| Pes1 (NP_001080557.1) | 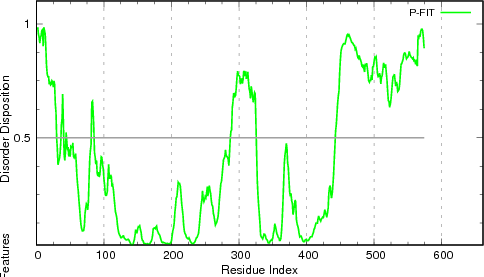 | MGGLEKKKYERGSATNYITRNKARKKLQLSLPDFRRLCILKGIYPHEPKHKKKVNKGSTAPRTFYLLKDIKFLLHEPIVGKFREYKVFVRRLRKAYGKREWDSVDRIRDNKPSYKLDHIIKERYPTFIDAVRDLDDALSMCFLFSTFPRTGKCHVQTIQLCRRLSVEFLNYVIDSRSLRKVFLSIKGIYYQADILGQTLTWITPYAFSHDHPTDVDYRVMATFTEFYTTLLGFVNFHLYQTLNLQYPPKLDSFSEVDLKSDGEDKYALETEVYMEKLAALSASLSRVIPSEPNDDTEVDEFPADPENAGLEEEQKRQLQEEEKHKSLFVGLKFFLNREVPRDALAFIIRSFGGEVSWDASVCIGATYNSTDPSITHHIVDRPSIQTQIINRYYLQPQWVFDCVNARLLLPVEDYFPGVLLPPHLSPFVHEKEGDYIPPEKLRLMAMQKGENLGLDEEDDDDDDDDEEEDDDDDEEEEDKKLRQLENKKVGQKNLNVKVTAGKVKVEDRTQVAEQEKNEEKRLAIMMMKKKEKYLYNKIMFGKKRKVREANKLALKRKAHDEAVKVERKKKAKKH | Granular component |

**Supplementary Table 1:** Disorder prediction and nucleolar localization. Column 1: Protein name. Column 2: Pondr-FIT prediction of intrinsically disordered regions(s) (IDR), with residue number on the X axis and predicted disorder (>0.5) on the Y axis. Column 3: primary sequence, with IDRs (as predicted by Pondr-FIT, Xue *et al*. 2010)[21] are highlighted in yellow and putative binding sites of Gar1 and Nhp2 that were mutated in this article are shown in red. Column 4: Nucleolar localization of the proteins, largely based on Lavering *et al.* (2022)[5].
