# Supplementary material for: Intrinsically disordered regions are not sufficient to direct the compartmental localization of nucleolar proteins in the nucleus
Source: PLoS Biol. 2023 Nov 9;21(11):e3002378. doi: 10.1371/journal.pbio.3002378 (PMC10662738; doi:10.1371/journal.pbio.3002378)
Supplement: S2 Table — This table shows the primers used to make the constructs shown in the paper and the fluorescent protein in each construct. They were made using Gibson Assembly or Q5 Site-Directed Mutagenesis as indicated in subheadings. *These were made only by amplifying the region of interest using the given primers using Q5 PCR and using the amplified region as a template for mRNA transcription. (DOCX) [file pbio.3002378.s004.docx]

Supplementary Table 2

| Constructs made using Gibson’s Assembly (NEB M5510AA) | | | | | | | |
| --- | --- | --- | --- | --- | --- | --- | --- |
|  | Fragment | | | Vector | | |  |
| Name | Forward Primer | | Reverse Primer | Forward Primer | | Reverse Primer | Fluorescent Protein |
| IDR-Ncl-GFP | ggatccacagccaccatggtgaaactagctaagg | | gttcttctcctttactcatctcaggaacattattttttggcat | atgagtaaaggagaagaac | | ggtggctgtggatccccc | mGFP5 |
| IDR-Ncl-Gar1-mCherry | ggatccacagccaccatggtgaaactagctaagg | | ccctcggaatgacatctcaggaacattattttttggc | tgccaaaaaataatgttcctgagatgtcattccgagg | | ggtggctgtggatccccc | mCherry |
| IDR-Ncl-Gar1-GFP | ggatccacagccaccatggtgaaactagctaagg | | ccctcggaatgacatctcaggaacattattttttggc | tgccaaaaaataatgttcctgagatgtcattccgagg | | ggtggctgtggatccccc | mGFP5 |
| IDR-Ncl-$\Delta$NGar1-mCherry | ggatccacagccaccatggtgaaactagctaagg | | ctcaggaacattattttttggcatttcttttttgcgttt | gagagtgttgttgaggttggggaatttatgc | | ggtggctgtggatccccc | mCherry |
| IDR-Ncl-Fbl-eGFP | ggatccacagccaccatggtgaaactagctaagg | | ggctgaatcctggcctcatctcaggaacattattttttggcatt | caaaaaataatgttcctgagatgaggccaggattcagc | | ggtggctgtggatccccc | eGFP |
| IDR-Ncl-$\Delta$NFbl-eGFP | ggatccacagccaccatggtgaaactagctaagg | | ctcaggaacattattttttggcatttcttttttgcgttt | ggagcaggaaggaaagtgatagtcgagcca | | ggtggctgtggatccccc | eGFP |
| IDR-Fbl-RFP | tccacagccaccaattcaaggcctctcgagatgaggccagga | | cggaggaggccattttccttcctgctcc | atggcctcctccgaggacgtcat | | ggtggctgtggatccccc | mRed |
| IDR-Fbl-eGFP | tccacagccaccaattcaaggcctctcgagatgaggccagga | | tctcctttactcattttccttcctgctccaaatccgcc | atgagtaaaggagaagaac | | ggtggctgtggatccccc | eGFP |
| Gar1-mRed | ggatccacagccaccatgtcattccgagggaga | | tcggaggaggccatacgccctcctcga | atggcctcctccgaggacgtcat | | ggtggctgtggatccccc | mRed |
| Gar1-mRed-Nb and Gar1M1-mRed-Nb | ACCGGCGCCGGAGGTGGAGGTGGAGCTatggcccaagttcagctggtt | | CCGCGGCCGCTCACCTACttaactaacggtaacttgcgtgccttgacc | ggatccacagccaccatgtcattccgagggaga | | ggtggctgtggatccccc | mRed |
| Nhp2P83A-mRed-Nb and Nhp2-mRed-Nb | accggcgccggaggtggaggtggagctatggcccaagttcagctggtt | | ccgcggccgctcacctacttaactaacggtaacttgcgtgccttgacc | gtaggtgagcggccgcggatctggttaccactaaacca | | ggccatagctccacctccacctccttaggcgc | mRed |
| Npm1-mRed-Nb | accggcgccggaggtggaggtggagctatggcccaagttcagctggtt | | ccgcggccgctcacctacttaactaacggtaacttgcgtgccttgacc | gtaggtgagcggccgcggatctggttaccactaaacca | | ggccatagctccacctccacctccttaggcgc | mRed |
| mRed-Nb | accggcgccggaggtggaggtggagctatggcccaagttcagctggtt | | ccgcggccgctcacctacttaactaacggtaacttgcgtgccttgacc | gtaggtgagcggccgcggatctggttaccactaaacca | | ggccatagctccacctccacctccttaggcgc | mRed |
| Constructs made using Q5 site directed mutagenesis (NEB E0554S) | | | | | | | |
| Name (template) | | Forward Primer | | | Reverse Primer | | Fluorescent Protein (s) |
| Nhp2P83A-RFP, and GFP (using the WT as a template) | | gctattgaggtttattgtcatatccctg | | | aagggtgtcaccagccattac | | mRed and mGFP5 |
| Gar1M1-mCherry, -mRed and -GFP | | gcagctgctgcagctgaatttatgcatcca | | | actctcaggaggaccttggtcatatcctcct | | mRed, mCherry and mGFP5 |
| ΔNNcl-GFP* | | tcgaaattaaccctcactaaagggatccacagccaccatgggcaacttaaactccac | | | actggccgtcgttttac | | mGFP5 |
| ΔNGar1-mCherry and GFP* | | tcgaaattaaccctcactaaagggatccacagccaccatggagagtgttgaggt | | | actggccgtcgttttac | | mCherry and mGFP5 |
| ΔNFbl-eGFP and mRed* | | tcgaaattaaccctcactaaagggatccacagccaccatggtgatagtcgagccaca | | | actggccgtcgttttac | | mRed and eGFP |

**Supplementary Table 2: Cloning Primers.** This table shows the primers used to make the constructs shown in the paper and the fluorescent protein in each construct. They were made using Gibson Assembly or Q5 Site-Directed Mutagenesis as indicated in subheadings. *These were made only by amplifying the region of interest using the given primers using Q5 PCR, and using the amplified region as a template for mRNA transcription.
